# Pairing pedagogical and genomic advances to prepare advanced practice nurses for the era of precision health

**DOI:** 10.1186/s12909-019-1542-x

**Published:** 2019-04-23

**Authors:** Elena Flowers, Margaret Martin, Hamza Abid, Sasha Binford, Lynda Mackin

**Affiliations:** 1 Department of Physiological Nursing, School of Nursing, University of California, 2 Koret Way, #605L, San Francisco, CA 94143-0610 USA; 2 Institute for Human Genetics, University of California, San Francisco, USA; 3 School of Nursing, University of California, San Francisco, USA

**Keywords:** Genomics, Technology, Advanced practice nurses, Pedagogy

## Abstract

**Background:**

Broadly accessible curriculum that equips Advanced Practice Nurses (APNs) with knowledge and skills to apply genomics in practice in the era of precision health is needed. Increased accessibility of genomics courses and updated curriculum will prepare APNs to be leaders in the precision health initiative.

**Methods:**

Courses on genomics were redesigned using contemporary pedagogical approaches to online teaching. Content was based on the Essential Genetic and Genomic Competencies for Nurses with Graduate Degrees.

**Results:**

The number of students enrolled (*n* = 10) was comparable to previous years with greater breadth of representation across nursing practice specialty areas (53% vs. 20%). Prior to the first course, students reported agreement with meeting 8% (3/38) of the competencies. By completion of the 3rd course, students reported 100% (38/38) agreement with meeting the competencies.

**Conclusions:**

Content on genomics sufficient to obtain self-perceived attainment of genomics competencies can be successfully delivered using contemporary pedagogical teaching approaches.

**Electronic supplementary material:**

The online version of this article (10.1186/s12909-019-1542-x) contains supplementary material, which is available to authorized users.

## Background

In 2015, the White House launched the Precision Health Initiative, directing healthcare towards a more targeted approach to preventing, understanding, and treating disease on the basis of genomic and other -omic information [1, 2]. Ahead of the Precision Health Initiative, in 2012 The American Nurses Association and International Society of Nurses in Genetics published Essential Genetic and Genomic Competencies for Nurses with Graduate Degrees. The competencies are organized into 38 individual items within seven themes across two domains (Table 1, Additional file 1: Table S1). Nurses, being at the forefront of healthcare delivery across settings and populations, are well positioned to become leaders in the delivery of precision health care [3, 4]. However, a major challenge is the very large workforce (i.e., 2.8 million currently licensed RNs) [5] that requires remedial education on current genomic technologies that are related to healthcare [6]. Prior studies conducted in multiple countries showed low levels of competency with genomics among nurses [7] and nursing faculty [8, 9]. The reasons for low levels of competency include a fundamental knowledge deficit, incomplete understanding of how genes effect health and disease, and long-standing misconceptions [10]. The lack of competency among faculty is particularly problematic given the magnitude of the nursing workforce. First targeting Advanced Practice Nurse (APN) educational programs may offer strategic advantages to overcoming this barrier. This approach will address two identified reasons for low competency levels by targeting nurses who develop a higher level of fundamental knowledge (i.e., knowledge deficit) and ability to interpret research findings (i.e., link the effect of genes on health and disease). Targeting this subset of the nursing workforce has the potential to enrich genomic knowledge for nurse leaders in order to further distill information across the large nursing workforce [11]. The goal is for all nurses to apply genomic information across the spectrum of clinical practice (e.g., obtaining family histories, appropriate referrals to specialists, interpretation of the results of genetic tests, and guiding treatment based on pharmacogenomics).

**Table 1:** Summary of essential genetic and genomic competencies for nurses with advanced practice degrees

Thematic Competency Area	
Practice	
Risk assessment and interpretation	
Genetic Education, Counseling, Testing, and Results Interpretation	
Clinical Management	
Ethical, Legal, and Social Issues	
Professional Responsibility	
Professional Role	
Leadership	
Research	

Online learning teaching approaches have become widely used throughout university settings. Online learning benefits both academic institutions through cost savings and students through flexibility to view course content on their own time and from distant locations. Summarized results of studies that evaluated online compared with or in addition to in-person learning approaches showed mixed results [12, 13]. Recent reports on best practices associated with online learning highlighted the increased effectiveness of hybrid approaches that emphasize the unique strengths of both online and in-person formats [12]. Evidence based hybrid approaches incorporate three key components: asynchronous access to online materials, online engagement of students through discussion boards, and faculty engagement [14].

A hybrid of online and in-person approaches to learning based on best practices derived from prior research is one tactic to address the challenge of educating the nursing workforce on current genomic knowledge and technology related to healthcare. This paper describes adaptation of in-person elective courses on genomics to incorporate contemporary online learning pedagogical approaches. The first goal was to increase accessibility of the courses across specialty areas within a Master’s of Science degree program for nurses. The second and third goals were for the courses to allow students to achieve competency in genomics [15] and for students’ satisfaction of the courses to be positive.

## Methods

### Conceptual framework

The hybrid approach to learning through a combination of online and in-person strategies was based on the Community of Inquiry conceptual framework, which focuses on process and iteration of curricular design [16]. This conceptual framework includes the key components of knowledge management for online learning including: availability of content anytime, anywhere (i.e., asynchronous); moderation and facilitation of open online forums; and faculty collaboration [16]. Students first obtained rote knowledge through online modules. This knowledge was reinforced first through engagement in online discussion boards and subsequently through opportunities to engage with the material collaboratively between faculty members and students during in-person meetings [16, 17]. This theory-based hybrid strategy enabled a process-oriented approach where students had the ability to learn and reflect on foundational content in an asynchronous manner, then utilize classroom time to ask questions and synthesize material that they had encountered prior to in-person class sessions [12, 14]. Student feedback was elicited both informally through discussion boards and in-person class sessions and formally through course evaluations for each term. This feedback was applied to each iteration of curriculum both during (i.e., through informal feedback) and upon completion of (i.e., through formal course evaluations) each term. This study was determined to be exempt by the Institutional Review Board at the University of California, San Francisco.

### Setting

Courses were offered in a graduate program for nursing that offers Master’s of Science and Doctorate degrees. All students were at the graduate level. The majority of the coursework is offered in-person with online courses primarily limited to electives. The genomics courses are open to students from all degree programs and specialty areas. The faculty of record for all courses was a doctoral prepared Registered Nurse and Clinical Nurse Specialist with a minor in education. Guest lecturers included a transdisciplinary cadre of licensed (i.e., Certified Genetic Counselor, Clinical Nurse Specialist) or doctoral prepared (i.e., Doctorate of Philosophy, Medical Doctorate) experts in the field of genomic fundamentals, clinical genomics, and bioethics.

### Structure

A hybrid format of both online and in-person content delivery was applied. Courses were 10 weeks in duration. The existing schedule included three academic terms in the standard academic year. Each academic term was referred to as a quarter associated with its respective season (e.g., Fall quarter). A three-course series was offered over the duration of the 2016–2017 academic year in sequential order. Students who elected to complete the minor focused on genomics were required to complete all three courses. A summary of the course objectives is shown in Table 2. The first course focused on fundamentals of human genomics, the second course focused on implications of genomics for APNs, and the third course focused on current examples of genomics in clinical practice across disease domains. Courses were scheduled in the evenings outside of the required coursework for all students. The first and second courses met in-person four times over 10 weeks and the final course was entirely online. Attendance was a component of student evaluation for courses with in-person meetings.

**Table 2:** Summary of Course Objectives

Course Sequence	Objectives
1	• Describe the structure and function of DNA, chromosomes and genes. • Describe patterns of inheritance, including Mendelian (e.g., autosomal dominant, autosomal recessive, and sex-linked transmission of genetic traits) and non-Mendelian (e.g., genetic heterogeneity and variable expression, genetic instability, mitochondrial and multifactorial inheritance); evaluate the scope, significance, and implications of the Human Genome Project and Genetic Information Non-Discrimination Act. • Describe the major mechanisms underlying genetic variation, including epigenetics, the techniques used to identify and quantify variation, and the molecular and clinical consequences of variation.
2	• Explain and discuss ethical, legal, and social issues in genomics. • Develop familiarity with the fundamental genomics competencies for registered nurses and advanced practice nurses; obtain and diagram a 3-generation family history and pedigree. • Explain and discuss issues related to diversity relevant to genomics. • Identify applications of genomics in clinical laboratories; discuss issues related to direct to consumer testing. • Analyze the differences and overlap between genomics in research and clinical practice. • Understand the Precision Medicine Initiative and implications for citizens, patients, and providers.
3	• Identify clinical applications of genomics across the lifespan. • Gain familiarity with current clinical implications of genomics for common diseases (e.g., cancer, neurological disease). • Articulate how to pursue a Certified Genetic Nurse credential. • Evaluate the research and resources relevant to providers and patients in your clinical specialty area.

### Assessment

#### Course satisfaction

Course evaluations were administered using a standardized school-wide online evaluation system (Additional file 2: Table S2**)**. Students in all courses offered by the school were asked to complete four questions pertaining to course satisfaction at the completion of each term. Anonymized results were returned to faculty members responsible for each course. Evaluation included a four-point Likert scale (i.e., Strongly Agree, Agree, Disagree, Strongly Disagree, or Not Applicable) and free text responses. Possible scores range from 1 to 4 with Not Applicable responses scored as zero and excluded from the overall score calculations.

#### Self-assessment of genomic competency

In order to assess sufficiency and appropriateness of the course content, students were asked to self-assess their agreement with having achieved the Essential Genetic and Genomic Competencies for Nurses with Graduate Degrees [11]. A 5-point Likert scale was administered via the online survey provider Survey Monkey (www.surveymonkey.com, San Mateo, CA). Response options included: Strongly Agree, Somewhat Agree, No Opinion, Somewhat Disagree, and Strongly Disagree. The survey was administered at four time-points, which included the first week of each academic term and the final week of the third academic term. The data collected in the first week of the second and third academic terms represented both knowledge gained in the prior term and baseline knowledge for the current term.

### Data analyses

Descriptive statistics were used to summarize course evaluations using Microsoft Excel version 15 (Redmond, WA). Responses for self-assessment of genomics competencies were scored using integers 1–5 then standardized to provide a range from − 1 to 1 using Microsoft Excel version 15 (Redmond, WA). Complete disagreement across all respondents corresponded to − 1. No opinion across all respondents corresponded to 0. Complete agreement across all respondents corresponded to 1.

## Results

### Course enrollment

A total of 10 students completed the genomics minor in the 2016–2017 academic year. In the 10 years prior to 2016–2017, the median number of students enrolled in each of the three courses that comprise the minor was 8 (mode 8; range 5, 12). Previous years’ class cohorts included students from 20% of the currently offered specialty areas (i.e., adult-gerontology, critical care, oncology) within the Master’s of Science degree program. The 2016–2017 cohort included students from 53% of the currently offered specialty areas (i.e., adult-gerontology, critical care, oncology, pediatrics, midwifery/women’s health, public health, and psychiatric/mental health).

### Course satisfaction

The school’s standardized course evaluations assessed three courses. Overall course satisfaction was 3.25 (range 3, 4) for the first course compared to school-wide average satisfaction for all courses in the term of 3.46. Overall satisfaction was 3.78 (range 3, 4) for the second course compared to school-wide average satisfaction of 3.46 for all courses in the term. Overall satisfaction was 3.50 (range 3, 4) for the third course compared to school-wide average satisfaction of 3.40 for all courses in the term.

Detailed text comments were provided by some respondents. For example, one student stated, “This class had a wonderful range of topics that expanded my understanding of Genomics in Health Care. This field is rapidly emerging and finding its way into both specialty and primary care - and I see this course as a requirement for all nurses.” Some comments provided constructive feedback for improvement. For example, one student stated “This was an adequate class with some good resources. I think it would have benefited from a few in person class meetings. The online discussions, while good, lacked some depth because we were not required to comment on every discussion and it’s always better to have a conversation face to face.” Another focus of some comments was the lack of connection between genomics fundamentals and clinical practice: “This was a nice introductory course to genomics and aspects of molecular biology. Perhaps I am looking for more clinical applications, but it seems like that will come next quarter…overall, I would have liked to see more connection with genomics and nursing in general.” Other comments highlighted that students’ learning occurred on a continuum, with not all concepts being mastered simultaneously. A comment provided in the second of the series of three courses stated: “This course really bridged nursing and genomics for me. Discussing the ethical/legal/social implications helped identify important barriers/concerns with this advancing technology…”.

### Competency self-assessment

**Fig. 1:**
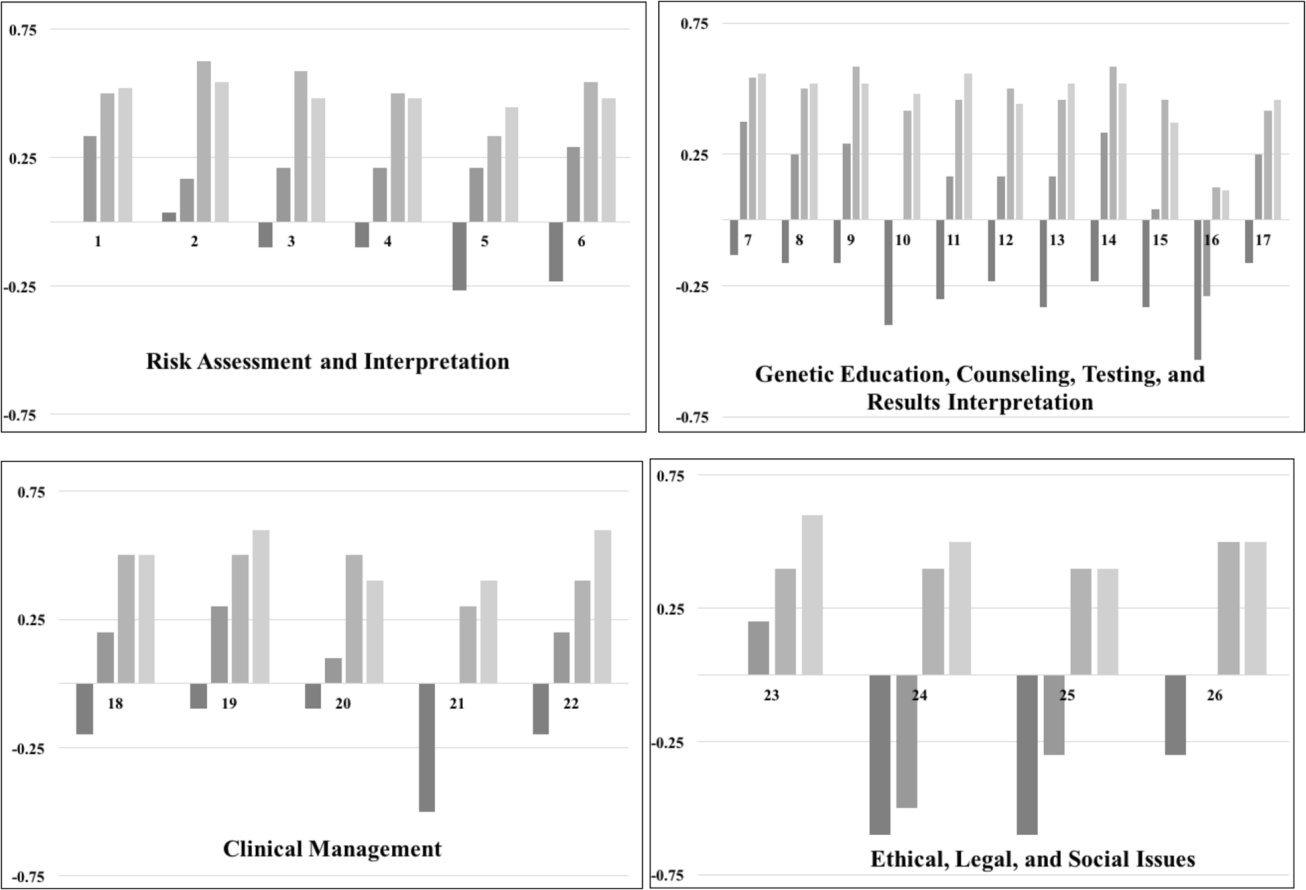
Students’ Self-Assessment of Professional Practice Competencies. Each panel shows self-assessed competency for each of the individual professional practice genetic and genomic competencies. Panels are organized by theme. Agreement with competency is shown on the Y-axis. Positive scores indicate agreement. Negative scores indicate disagreement. Scores of zero correspond to the “No Opinion” response option. Competency number is shown on the X-axis. See Table 1 for individual competencies. Assessment time points are from left to right for each competency with the darkest bar representing the initial assessment at the beginning of the first course and the lightest bar representing the final assessment at the completion of the third course

**Fig. 2:**
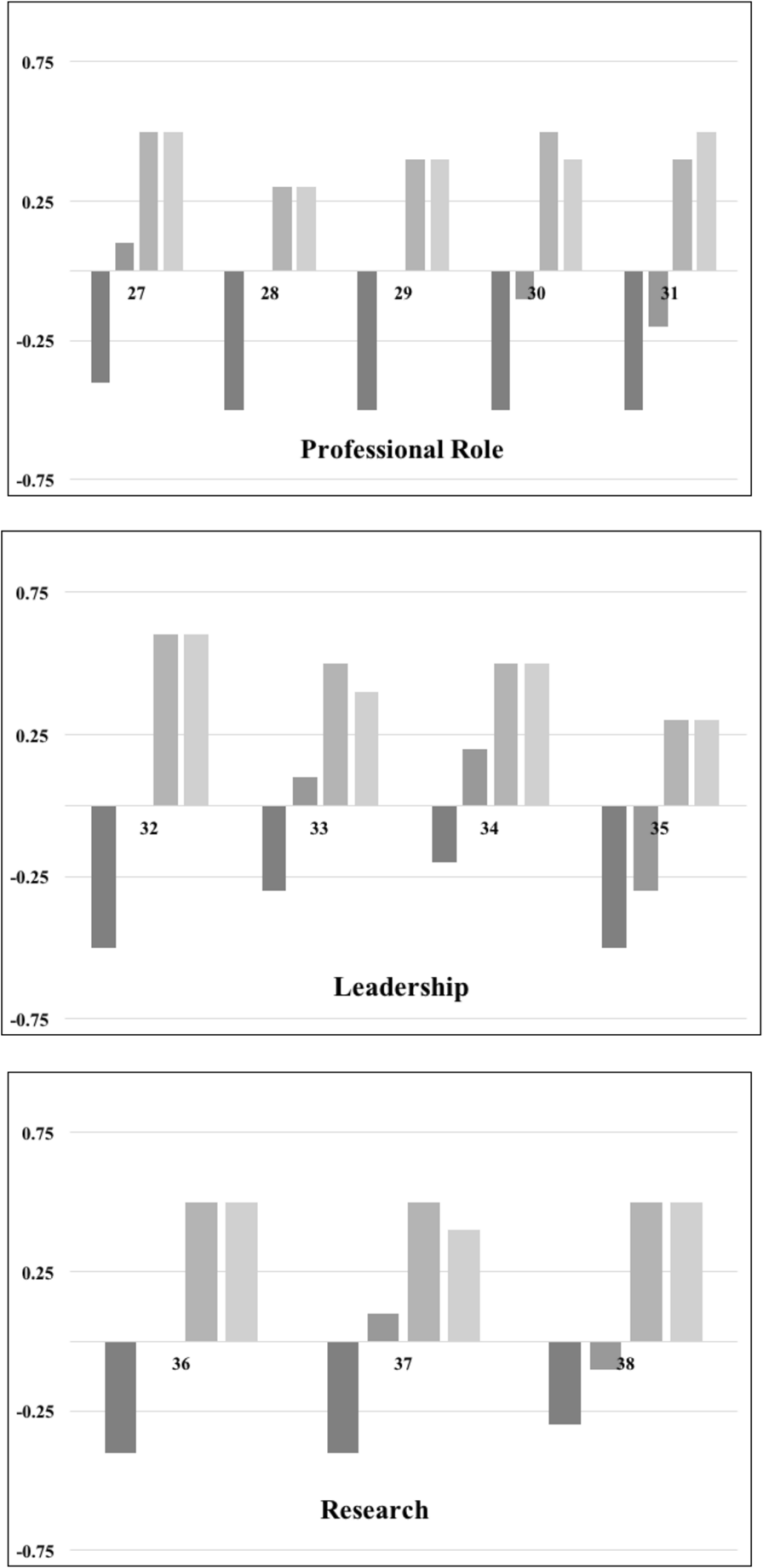
Students’ Self-Assessment of Professional Responsibility Competencies. Each panel shows self-assessed competency for each of the individual professional responsibility genetic and genomic competencies. Panels are organized by theme. Agreement with competency is shown on the Y-axis. Positive scores indicate agreement. Negative scores indicate disagreement. Scores of zero correspond to the “No Opinion” response option. Competency number is shown on the X-axis. See Table 1 for individual competencies. Assessment time points are from left to right for each competency with the darkest bar representing the initial assessment at the beginning of the first course and the lightest bar representing the final assessment at the completion of the third course

Students self-evaluated perceived agreement with having achieved 38 individual competencies within 7 themes across two domains (Table 1). Average agreement across all individual competencies at the beginning of the first course was − 0.3 (range − 1, 1). At beginning of the second course, average agreement increased to 0.1. At both beginning and completion of the third course, average agreement increased to 0.5. Upon completion of the second course, all students reported agreement scores with all of the 38 individual competencies (Fig. 1, Fig. 2).

## Discussion

Advances in technology have facilitated emerging genetic tests, genetic therapies, and reconsideration of how diseases are classified that directly impact clinical practice for nurses. As evidenced by the Institute of Medicine Report on Precision Medicine, [18] the Essential Genetic and Genomic Competencies for Nurses with Graduate Degrees, [11] and other consensus statements [19], application genomic technologies in health care and the pursuit of precision health are relevant across the age span, care settings, and disease areas. In parallel, APNs caring for patients across the age span (e.g., pediatrics, adult-gerontology), in diverse care settings (e.g., acute/critical care, public health), and across all specialty areas (e.g., oncology, mental health) urgently need education about the clinical implications of current genomic technologies.

In order to address challenges related to educating APNs about genomics, a series of three in-person courses were adapted to a hybrid online and in-person format. Through application of evidence based pedagogical approaches [14, 16], students first viewed material on fundamental concepts related to genomics (e.g., structure and function of DNA, family history and pedigrees, genetic testing, pharmacogenomics). Students were provided with self-paced, graded quizzes in order to assess learning. Then in-person class sessions were designed to supplement and enrich online presentation of materials and forum discussions with greater depth on complex topics (e.g., patterns of inheritance, ethical, legal, and social issues).

Completion of all three courses conferred APN students with a minor in genomics. The challenges overcome by this pedagogical approach included management of schedule conflicts and academic credit load across specialty areas within a Master’s of Science degree program for nurses. This approach was successful at the three pre-specified goals: increasing accessibility of the courses to a broader range of specialties, achievement of competencies related to genomics, and acceptable student satisfaction with the courses.

Evaluation of students’ free text comments for course satisfaction showed overall positive comments for the genomics courses. Importantly, students gained an appreciation for the importance of genomics for APN practice. In the example provided in the Discussion section, students highlighted the breadth of topics and critical importance of the topic area as strengths of the courses. Critiques of the courses had a specific recurrent theme of wanting more in-person class sessions and/or more engagement in the online learning forum. This is a common challenge for online learning environments. Future evidence-based modifications to the courses could include synchronous video sessions and greater moderation of discussion boards by the instructor and/or teaching assistant in order to increase student engagement within an online learning forum [14].

Course evaluation scores showed students gave primarily “somewhat agree” ratings for all three courses with some “somewhat disagree” and some “strongly agree” responses. The lowest overall scores were for the first in the series of courses, which focused on fundamentals of human genomics (Table 2). Students’ free text comments indicated frustration with the lack of connections between genomics fundamentals and nursing practice. The courses are structured so that the first in the series focuses entirely on fundamental concepts related to genomics and not at all on clinical practice. One possibility is that this connection is then made in the second and third courses by building on the foundational knowledge gained in the first course. Repeated assessment of whether students felt genomic fundamentals inform their clinical practice across all courses suggested that this linkage became more clear after the first course. The highest overall evaluation was for the second course, which focused on implications of genomics for APNs (Table 2). Based on students’ free text comments, the frustrations from the prior term were decreased and the linkages between genomics and nursing practice were clarified.

At baseline, students consistently reported having achieved competency with two of the 38 individual competencies (Fig. 1). The first was competency #1: Identify clients with inherited predispositions to diseases as appropriate to the nurse’s practice setting. A fundamental competency for Registered Nurses is to obtain and three-generation family history and construct a pedigree [15]. The observation of high agreement with competency #1 suggests pre-licensure programs are meeting this competency, and nurses enter APN programs with sufficient training in this area. This competency also showed the greatest agreement upon completion of all three courses, with substantial increase in the agreement score compared to baseline (Fig. 1).

The lowest agreement scores at baseline and after completion of all three courses was competency #16: Select appropriate genetic/genomic tests and/or studies. Overall, students went from an average score within the disagreement range to an average score of agreement (Fig. 1). Given the current landscape and pace of genomic technological advances, mastery of this competency within general courses on genomics targeting a broad range of APN specialties is not a realistic goal. Rather, courses should provide education about principles of test selection (e.g., clinical validity, clinical application) that students can use as a foundation to build knowledge about resources and tools specific to their specialty areas.

Overall, the students rated higher competency within the professional practice domain compared to the professional responsibility domain (Fig. 1, Fig. 2). Another competency with low agreement at the completion of the coursework was #35: Influence health policy at the local, state, national, and international levels related to genetics/genomics. A possible future modification of the coursework is to create an assignment to draft a letter to a local or national elected representative that advocates for or against legislative action related to genomic policy.

## Limitations

The assessment and evaluation of courses on genomics is limited to a single offering of each of three courses over one academic year. Integration of data from additional years and larger and more heterogeneous samples will increase the generalizability of the conclusions to a broader population of students. Evaluation of learning was done by student self-assessment of competency. Future evaluations should utilize the Genomic Nursing Concept Inventory, which measures genomics knowledge [20].

## Conclusions

Clinical implications that result from recent advances in genomics technology and a focus on delivery of precision healthcare have created a need to develop a broadly accessible curriculum that equips APNs with knowledge and skills to apply genomic tools in practice. Courses delivered using pedagogically contemporary approaches, including hybrid course models, successfully accomplished three educational goals. A greater breadth of students were able to access the courses, students reported satisfactory evaluations of the courses, and students achieved competency with genomics in clinical and professional practice domains.

## Additional files

Additional file 1: **Table S1.** Shows each competency, categorized by theme. (XLSX 11 kb)

Additional file 2: **Table S2.** Shows the response options for assessment of satisfaction with the course. (XLSX 8 kb)

## Abbreviation

APN: Advanced Practice Nurse
